# Bridging the gap: A qualitative study on veterans’ experiences with systemic barriers and support during transition

**DOI:** 10.1371/journal.pone.0361285

**Published:** 2026-11-06

**Authors:** Polly Ingram, Amanda Mele, Christina M. Lazar, Kristin Mattocks, Megan Kelly, Sara Edmond, Marc I. Rosen, Adrienne Weede, Jennifer Perez, Steve Martino

**Affiliations:** 1 VA Connecticut Healthcare System, West Haven, Connecticut, United States of America; 2 Yale University School of Medicine, New Haven, Connecticut, United States of America; 3 VA Central Western Massachusetts Healthcare System, Leeds, Massachusetts, United States of America; 4 University of Massachusetts Chan Medical School, Worcester, Massachusetts, United States of America; 5 VA Bedford Healthcare System, Bedford, Massachusetts, United States of America; 6 Department of Veteran Affairs, Washington, D.C., United States of America; Uniformed Services University: Uniformed Services University of the Health Sciences, UNITED STATES OF AMERICA

## Abstract

Many veterans face complex challenges during the transition from military to civilian life, particularly when navigating healthcare and benefits systems. This qualitative study examines how recently separated veterans with service‑connected musculoskeletal disorders perceive and navigate systemic barriers within Department of Defense and Veterans Affairs transition and healthcare systems. Semi‑structured interviews were conducted with 20 veterans within one year of separation who had filed a musculoskeletal service‑connection claim. Using a rapid qualitative analytic approach, interviews captured experiences with the discharge process, transition programming, and efforts to access healthcare and benefits. Analysis identified four major themes: the interaction of physical, psychological, and bureaucratic stressors during discharge, particularly salient for those managing musculoskeletal conditions; varied and often critical views of transition programming, which many perceived as generic and misaligned with individual needs; systemic barriers to care and benefits, including delays, fragmented processes, and cultural norms that discourage disclosure; and recommendations emphasizing continuity of care, case management, and emotional support to address identity disruption and loss of purpose. Overall, the findings highlight persistent structural and cultural obstacles that complicate transition and hinder early engagement with services. Improved coordination, continuity‑oriented supports, and psychologically responsive programming may reduce these barriers. Further research is needed to evaluate scalable models that enhance early post‑separation outcomes.

## Introduction

The period following military separation is one of heightened vulnerability, marked by increased risk for psychological distress, disruptions in social and occupational functioning, and ongoing physical health challenges [1]. Research indicates that recently separated service members are up to 2.5 times more likely to die by suicide compared to the general population [2], and rates of post-traumatic stress disorder (PTSD) and depression remain higher than their active-duty counterparts [3–6]. Social and occupational reintegration can also be difficult, with many veterans facing interpersonal strain, role disruption, and increased risk of homelessness and unemployment [1]. Early problems with reintegration tend to persist over time, and these disruptions may partially account for why veterans face a substantially higher risk than civilians for mental health conditions, suicidal behaviors, and substance use disorders [3,7].

In addition to these challenges, veterans with service-connected musculoskeletal disorders, including chronic back pain, joint injuries, and mobility impairments, face unique and often underrecognized barriers [8]. Musculoskeletal conditions represent the largest category of service‑connected disabilities, accounting for 38% of all VA disability compensation claims [9]. These injuries can significantly impair quality of life and require ongoing medical care, pain management, and rehabilitation [10]. However, the transition from military service to VA healthcare for veterans with musculoskeletal disorders remains poorly understood, particularly how these individuals navigate systems of care and perceive the support available to them.

In recognition of the complexity of the transition process, federal agencies have developed a myriad of programs and services to support service members before, during, and after separation (see the 29 programs listed in Table 1). In an attempt to provide some framework for understanding the many options, the Transition Assistance Program (TAP), a joint initiative led by the Department of Defense (DoD), Department of Veterans Affairs (VA), and Department of Labor (DOL), with contributions from the Department of Education, Small Business Administration, and others, was established [11]. TAP provides modular, outcome-focused programming, including a required week-long “course”, that includes financial planning, career readiness, and benefit navigation [11]. The DoD also oversees programs for post-separation, such as the *in*Transition program, which aims to ensure follow up mental health care and treatment in coordination with the VA Solid Start and VA Liaison and Post-9/11 Transition and Case Management Programs. These VA programs monitor veteran outcomes, provide early clinical care coordination and screening for high risk factors, and encompass individualized case management to ensure continued access to resources. Veterans Service Organizations (VSO) and peer networks further supplement institutional support.

**Table 1:** Separation and transition-related resources and programming.

Pre-Separation					
Program / Resource	Agency	Year Established	Focus	Eligibility / Access	Description
Initial Counseling	DoD	2019	Career Planning, Case Management	Transitioning service members beginning TAP (up to 24 months before retirement / 12 months before end of service	Starts transition planning and Individual Transition Plan creation.
Pre-Separation Counseling (DD 2648)	DoD	1991; updated in 2011	Benefits, Career, Education	Required by all separating service members (no later than 365 days before end of service)	Overview of benefits and transition steps.
Service-Specific Branch Transition Programs	DoD	1991	Transition Counseling, Workshops	Transitioning service members beginning TAP process based on service policy	Each branch runs its own TAP variant to deliver DoD TAP curriculum locally.
TAP Core Curriculum & Tracks	DoD	2014	Employment, Education, Financial	TAP-enrolled service members	Mandatory training & career/education tracks.
Capstone Preparation	DoD	2011-2014	Case Management, Readiness	Transitioning service members prior to final Capstone	Prepares for Career Readiness Standards verification.
**Separation Window**					
**Program / Resource**	**Agency**	**Year Established**	**Focus**	**Eligibility / Access**	**Description**
SkillBridge	DoD	Piloted in 2011; formalized in 2014	Employment, Apprenticeship	Transitioning service members final 180 days with command approval	Civilian internship/training while in service.
Capstone Verification	DoD	2011-2014	Case Management, Readiness	All transitioning service members before end of service	Confirms TAP completion & readiness.
VA TAP Briefings	VA	1991; redesigned in 2011, 2019	Benefits, Medical	Transitioning service members in TAP	Full walkthrough of VA services, benefits & systems.
PCPG / Chapter 36 Counseling	VA	2011	Career Planning	Transitioning service members within 6 months pre-separation or veterans	Personalized career & education advising.
VA Healthcare Pre-Enrollment (optional)	VA	2011-2014	Medical	Transitioning service members planning to separate	Start VA healthcare enrollment pre-ETS.
BDD Disability Claim Prep	VA	2007	Benefits	Transitioning service members 180–90 days pre-separation	File disability claim to speed VA decision post-ETS.
VSO Claims Assistance (Disabled American Veterans (DAV)/Veterans of Foreign Wars (VFW)/American Legion	VSO	1970−80’s; expanded in 1991	Claims & Benefits	Any service member prepping to separate	Help prepare VA claims and evidence.
**Post-Separation**					
**Program / Resource**	**Agency**	**Year Established**	**Focus**	**Eligibility / Access**	**Description**
VA Solid Start	VA	2019	Benefits, Case Management	All new veterans (0–12 months)	Proactive phone outreach to connect to benefits.
VA Healthcare Enrollment	VA	1991	Medical	Eligible veterans	Primary/specialty/mental healthcare.
Vet Center Services	VA	1979	Mental Health, Community	Combat/Military Sexual Trauma-qualified veterans	Confidential counseling & support.
VA Disability Compensation	VA	Modern structure established 1950−90’s	Benefits	Any veteran with service-connected conditions	Compensation & rating decisions.
VR&E (Chapter 31)	VA	1943; modern structure established 1980	Employment, Education, Case Management	Veterans w/ service-connected disabilities	Vocational rehab, training, job placement.
GI Bill / Education Benefits	VA	1944; updated in 1984, 2008	Education	Eligible veterans	Education funding: tuition, housing, exams.
HUD-VASH / SSVF Housing Support	VA	1992; SSFV in 2011	Housing, Case Management	Homeless/at-risk veterans	Housing vouchers & case management.
VA Veteran Sponsorship Initiative (VSI)	VA	2022-2023	Mentorship, Case Management	New veterans (participating regions)	1:1 transition guidance (jobs, housing, benefits).
VSTAGP (VA-funded nonprofits)	VA	2021-2023	Employment, Case Management	Veterans & spouses	Job training & transition support via VA grants.
VA Liaison Program	VA	2003	Care Transitions,Healthcare, and Coordination	Transitioning service members	Individualized clinical care coordination from DoD to VA health care facilities and/or from VA’s public-private partners to VA facilities nationwide.
Post-9/11 Military2VA (M2VA) Case Management	VA	2007	Care Transitions, Healthcare Navigation, Clinical Case Management	Transitioning service members and Post-9/11 era veterans	DoD to VA clinical care transitions and coordination as well as high risk screening and ongoing case management..
Wounded Warrior Project - Warrior Care Network (WCN)	VSO	2015	Mental Health	Post-9/11 era veterans with trauma- and injury-related needs	Intensive clinical treatment for complex MH challenges. VA Liaisons for Healthcare are onsite at WCN sites to provide direct clinical care coordination and access to VA health care facilities.
Wounded Warrior Project – Warriors to Work	VSO	2006	Employment	Post-9/11 era veterans	Career coaching & job placement support.
Team Red White and Blue	VSO	2010	Community, Wellness	All veterans & transitioning service members	Community connection & wellness events.
Team Rubicon	VSO	2010	Service, Purpose	All veterans	Mission-based community service & reintegration.
American Legion / Disabled American Veterans (DAV)/Veterans of Foreign Wars VFW) Service Officers	VSO	1950’s; expanded in 1970–1990’s; formalized in 1991	Benefits, Claims	All veterans	Ongoing claims representation & benefit navigation.
Code of Support Foundation Case Management	VSO	2010	Case Management, Crisis Support	Veterans & families	Navigate access to benefits, services & address multi-system barriers.

Despite the availability of structured transition programs, veterans continue to encounter significant challenges understanding and navigating these systems. Recent government reports have highlighted systemic shortcomings in the implementation and evaluation of transition services [12–15]. Although DoD requires service members to begin TAP at least one year before separation, most service members start later, often because they initiate TAP close to the end of an enlistment contract or because timelines are compressed in cases of medical separation. More than half begin fewer than nine months prior [13]. Additionally, while workshops may be waived when service members are assessed as already prepared, the high rate of waivers limits consistent exposure to core program content [13]. Furthermore, programs such as DoD *in*Transition and warm handovers are underutilized or poorly tracked, with over 70% of eligible service members not contacted [16]. VA outreach programs to veterans who have left active duty such as VA Solid Start have shown promise, with contacted veterans significantly more likely to engage with VA healthcare, yet outreach remains inconsistent, especially among younger veterans and those facing housing instability [13]. Moreover, the proliferation of transition-related programs across federal agencies has led to fragmentation and confusion, with many veterans reporting difficulty identifying which services are relevant or accessible to them [17]. These findings underscore the need to examine how veterans perceive, engage with, and are impacted by these systems.

This study seeks to explore veterans’ experiences during the transition from military to civilian life, with particular attention to the systemic barriers encountered by those managing service-connected musculoskeletal conditions. While there is existing research on veterans’ outcomes and service utilization, there is limited information from veterans about how they experience the transition and what specific systemic obstacles or facilitators they encounter. This gap means that policies and program improvements may not fully address the real challenges veterans face during their transition, potentially leading to unmet needs or ineffective support structures. Understanding veterans’ perspectives is critical to identifying barriers that may not be captured in administrative data or program evaluations.

## Materials and methods

### Study approach and design

This paper presents a secondary analysis from a qualitative study examining post-separation experiences of recently discharged veterans [18]. This current analysis examines veteran’s perceptions of systemic barriers and facilitators within VA programs and healthcare navigation. We used a deductive rapid qualitative analysis approach, organizing data collection and analysis around predefined theoretical constructs while remaining open to emergent insights. Rapid methods emphasize structured summaries, matrix-based comparison, and team-based interpretation rather than full transcription coding, making them well-suited for applied health research where timely findings are needed [19–22]. Our methodological orientation was pragmatic, emphasizing practical problem‑solving and actionable insights. This orientation guided the deductive structuring, the use of structured summary templates, and the prioritization of streamlined but rigorous analytic procedures.

### Participants and recruitment

Participants were recruited from a VA study focused on musculoskeletal service-connected conditions and transition experiences [18,23]. Inclusion criteria required that participants had separated from military service within the past year and had filed a musculoskeletal service‑connection claim. Recruitment and consent procedures followed VA IRB-approved protocols. Because interviews were conducted virtually, the VA Connecticut Healthcare System IRB approved a waiver of written consent and permitted verbal consent (#1752660). Research staff obtained verbal consent using an IRB‑approved script and audio‑recorded each consent interaction, labeling the recording with the participant’s name and date. The audio recording served as documentation of consent and was stored separately from all study data on secure VA servers.. We used purposive sampling from the parent study to ensure variation across service branch, sex, race/ethnicity, and rank.

Recruited between March and May 2025, 36 veterans were invited to participate, 7 did not attend scheduled interviews, 2 declined participation, and 7 did not respond to outreach attempts. The final sample included 20 veterans (55% male). Ages ranged from 23 to 45 years (M = 30.9, SD = 6.5). Most participants identified as White (50%), followed by Black (25%), Asian (15%), and Native Hawaiian or Pacific Islander (5%). Fifteen percent were Hispanic or Latino. Participants represented all major branches of service: Air Force (25%), Army (25%), Marine Corps (30%), and Navy (20%). They had served an average of 8.9 years (SD = 6.8; range = 1–22 years). Time since separation averaged 8.3 months (SD = 3.2; range = 3–12 months). Thirty-five percent had separated within 7 months and 65% within 8–12 months. At discharge, 65% were enlisted (E3 to E5), 15% were senior enlisted (E6 to E8), and 20% were officers (O3 to O7).

### Interviews and data collection

Semi-structured interviews were conducted virtually via Microsoft Teams between March and May 2025, lasted approximately 45–60 minutes, and were audio-recorded and transcribed. Interviews were conducted by the lead author, accompanied by a second analyst present to take detailed field notes using the standardized template. Participants had no prior relationship with the interviewer and were informed of the interviewer’s role and the study purpose. The interview guide was pilot‑tested within the parent study to ensure clarity and relevance. No repeat interviews were conducted, and transcripts were not returned to participants due to the rapid analytic design. The interview explored veterans’ service experiences, transition challenges, and post-military functioning. For additional details, see the previously published report (see S1 Appendix for interview guide) [18].

Immediately following each interview, the interviewer and note taker reviewed and refined the summary, clarified unclear responses, and documented emerging observations as a quality assurance step. Summaries were compiled into a matrix in Microsoft Excel, with rows representing participants and columns aligned with interview questions. Audio timestamps preserved linkage to raw data for validation. This matrix enabled cross-case comparison and facilitated identification of thematic patterns. All data (audio files, transcripts, summary templates, and analysis matrices) were stored on VA‑secure servers with encrypted drives and encrypted files.

### Analysis

Analysis focused on experiences related to discharge processes, transition programming, healthcare navigation, and system‑level recommendations. Following data collection, the lead author reviewed the matrix to identify initial patterns, which were refined through iterative consultation with a senior qualitative mentor and consensus meetings with two additional analysts. These meetings clarified theme boundaries and ensured consistent interpretation. Member checking was not conducted due to the rapid analytic design. We used Microsoft Excel to manage the summary matrix, conduct cross‑case comparisons, and maintain an audit trail linking summaries to raw data timestamps. As is typical in rapid qualitative analysis, thematic sufficiency in the parent study was judged by matrix completeness rather than code-level saturation. Later interviews consistently aligned with existing domains and did not add new ones, indicating adequate coverage of the deductive framework. The present secondary analysis relies on that established sufficiency. To enhance trustworthiness, we incorporated triangulation of field notes with transcripts, team‑based consensus, timestamp‑based verification, and documentation of analytic decisions. Reflexive discussions were held throughout analysis, and the lead interviewer maintained notes regarding potential assumptions and their influence on interpretation.

To ensure rigor, the Planning for and Assessing Rigor in Rapid Qualitative Analysis (PARRQA) framework was applied [24]. The original study adhered to all PARRQA domains, including rigorous design, systematic data collection, summary template development, matrix analysis, and team-based synthesis [25]. This secondary analysis followed the same procedures for data handling and interpretation as in the primary study [25]. We further ensured alignment with Consolidated Criteria for Reporting Qualitative Research (COREQ) [26], the Standards for Reporting Qualitative Research (SRQR) [27], and the National Institute for Health and Care Excellence (NICE) qualitative reporting guidance [28]. A detailed mapping of these frameworks to study procedures is provided in the supplementary materials (S2 Appendix).

## Results

This qualitative study explored veterans’ experiences transitioning from military to veteran health systems, with a focus on systemic barriers and support mechanisms. Through thematic analysis, four major themes emerged.

### Theme 1: Discharge as a Difficult Process Marked by Physical, Mental, and Institutional Strain

Veterans consistently described the discharge process as a period of heightened vulnerability, shaped by physical injuries, mental health challenges, and institutional inefficiencies. For many, the discharge process set the tone for their post-separation experience with health and benefits systems, as delays, unclear procedures, and inconsistent communication compounded the emotional and physical toll of leaving service. These early systemic barriers often disrupted continuity of care and contributed to a sense of abandonment during a critical life transition (see Table 2 for supporting quotes).

**Table 2:** Theme 1: Discharge as a difficult process marked by physical, mental, and institutional strain, subthemes with supporting quotes.

Subtheme	Frequency (n, %)	*Supporting Quotes*
**1. Physical health as primary or contributing driver of discharge**	13 (65%)	*I tore my ACL in the Marine Corps. I had surgery after deployment. I postponed it just so I could deploy. It was the third time I've torn my ACL in my life. Like it's Marine Corps, the most physical out of all the military branches which is great, and I've been competitive in, but I just don't feel like I should be pushing my body to that point where it continues to break.-* Marine Corps veteran, female, age 32
		*I had gone to the doctor because I had been battling my back and my leg. I had this nasty bulging disc in the lower part of my back and sciatica in my leg. It was severely limiting me on a daily basis, and I realized that I probably needed to get seen for it... I would say [injury] was a major factor [in my separation]. Being a diesel mechanic, being a soldier, and doing intense PT – the workouts almost require you to hurt yourself. You hurt yourself if you don’t do them the way they want.* - Army veteran, male, age 25
**2. Mental health strain contributing or coinciding with discharge**	18 (90%)	*Even though I had physical ailments, I was struggling mentally, really depressed and anxious. At first, they said it didn’t seem like I’d gotten any help, so I talked to a counselor a few times, which helped a little, but I still felt like I needed to leave. They recommended working out, which I was already doing, but previous injuries came back, starting in my feet, then moving to my knees and hips. It was an endless cycle. If you’re not active, you feel down because you’re not being productive. It was either workout and injure myself more, or don’t and feel depressed and stuck inside. I went to mental health and kept going back, saying I felt worse. Eventually, a social worker said they’d discharge me for not adjusting to military life, but the note said I wouldn’t get disability for it.*-Navy veteran, female, age 32
		*At six years, I pretty much decided to call it, mostly because of this last unit I was in. It just was so high stress. It was very negative. I did start to have anxiety – severe symptoms, like physical symptoms. So, for my own mental health, I was just basically like, this probably isn't the right fit.* -Marine Corps veteran, female, age 32
**3. Bureaucratic delay and lack of transparency surrounding discharge process**	11 (55%)	*It was honestly annoying because it was so prolonged. It's hard to plan your life when you're getting out, and with me being the type of person I am, I prefer to be proactive, so I made a lot of arrangements. I started my process in March of 2023, but I didn’t get out until July of 2024. I was trying to be proactive, thinking, well, they told me I should be out of the Navy by September or October at the latest. I had already paid money to start school. Then they said I shouldn’t have made all these arrangements for life outside the Navy, even though they were putting me out because of my injuries. But I still had to work for them in the meantime. I know everybody’s case is different, but if you know you’re going to be putting me out because of my injury, what is the holdup? I can’t start my life until you close the chapter on this. I can’t close it – you have to close it for me.* -Navy veteran, female, age 31
		*The entire discharge process was kind of a nightmare. Nobody who's currently in the military knows what the process for getting out is fully. Everybody knows that their role is in somebody's discharge, but nobody can tell you like what the next steps are. It was about a year of me running around asking everybody: What do I need to do next? Where do I need to go? What paperwork does this? Who does this go through? It was just a mess. You don't know until you're out.* -Navy veteran, male, age 29

Many participants identified service-related injuries and declining physical health—most often chronic musculoskeletal pain, mobility limitations, and cumulative wear‑and‑tear—as primary factors driving their separation, often resulting in premature discharge. Mental health concerns also played a significant role, with veterans describing how the high-stress, high-tempo nature of military life contributed to conditions such as anxiety, depression, and PTSD. As veterans started the discharge process, they often faced delays and little clarity about next steps or the status of their documents. For many, the timeline extended well beyond initial expectations, often lasting over a year, and was marked by failure to provide clear, timely, and coordinated guidance. These systemic challenges prolonged the transition, setting a trajectory of uncertainty and limited support during a critical period of adjustment.

### Theme 2: Mixed Experiences with Transition Support

Veterans’ experiences with transition support programs varied widely, reflecting both the strengths and limitations of current systems. While some participants found value in structured programming and tailored resources, many described the standard offerings as generic, poorly timed, and difficult to navigate. These inconsistencies suggest that while transition assistance programs aim to provide comprehensive support, their effectiveness is often undermined by a lack of personalization and coordination. The findings highlight a need for more adaptive, veteran-centered approaches that account for individual circumstances and timing (see Table 3 for supporting quotes).

**Table 3:** Theme 2: Mixed experience with transition support, subthemes with supporting quotes.

Subtheme	*Frequency (n, %)*	*Supporting Quotes*
**1. Generic, checklist-based programming that lacked personalization**	14 (70%)	*The military has TAPs (Transition Assistance Program), but as a person who had gone through college and had a master’s, it was almost worthless. There was no point for me to do that. It was career decisions and financial planning, stuff like that. But outside of that, they didn’t offer anything. They gave me a checklist and said, “This is where you need to process at, figure it out, and be on your way.” I had already planned for my discharge so, for me, it was like, I did all this already, and I’m just doing it to check a box.* -Air Force veteran, female, age 35
		*They had a TAP (Transition Assistance Program) class on base. Those classes kind of annoy me because a lot of class time gets spent answering basic questions. I’m also in a unique situation: I knew I was going to be a stay-at-home mom. I had no plans to work, so I didn’t need the interview steps, how to dress for a job, or any of that. I had already done the research on the financial side, so I was like, do I really need to sit in a class for eight hours just to be told how to write a budget?* -Army veteran, female, age 42
**2. Confusing, disorganized, or poorly timed process**	10 (50%)	*There’s a program called TRS (Transition Readiness Seminar) where you go to a two-week course. They teach you how to make a LinkedIn and talk about the process of getting out. But after getting out, it feels totally inadequate, just like an administrative checkmark. Like, “Cool, we did our thing, so he’s good.” I had to do it before deploying, instead of two months before separation. I did it eight or nine months before getting out, then deployed, came back, and started processing out of the Marine Corps almost immediately. So, I can’t say it was fresh in my mind.* -Marine Corps veteran, male, age 26
		*There was a lot of good information in the TAPs (Transition Assistance Program) classes. It was just a matter of understanding the order of things and where your paperwork needed to be at any given time. I remember week after week of bouncing between medical and admin, trying to figure out who needed my medical paperwork and where it needed to go so admin could write my DD214. Admin said I needed other things signed off on my medical paperwork before I could turn it in, but medical said the exact opposite. It was just constant back-and-forth with no clear answers.* -Navy veteran, male, age 29
**3. More effective when tailored, or supported by alternative sources**	17 (85%)	*The biggest thing I went through was the Soldier for Life Transition Assistance Program (SFL-TAP). I was able to get into the week-long E8s and above program, so everything was laid out for us. We were catered to; it was mostly higher-ranking soldiers and officers. We had a ton of resources and materials, and different people brought in various information. SFL-TAP really set my foundation for retirement preparation.* -Army veteran, male, age 41
		*If you're a reservist coming off deployment, they also offer a school-related program called the Yellow Ribbon Program, which was extremely helpful. It's mostly for the family, so they know how to support your transition, mentally, emotionally, and jobwise. They learn the process too. That was especially helpful for me because I was connected with a lot of different mental health specialists, third-party programs, and Wounded Warrior reps – people who could actually help me. I would have never known about them if they hadn’t all been there.* -Marine Corps veteran, male, age 28

Most participants expressed neutral or mixed feelings toward the standard week-long TAP curriculum course, acknowledging its coverage of career planning, financial literacy, and VA-related resources. Some veterans found this content helpful, particularly in orienting them to civilian systems. However, others described the program as overly generic and insufficiently responsive to individual needs, characterizing it as a “one-size-fits-all” approach that lacked relevance to their specific circumstances. In addition to content concerns, veterans frequently described the timing of transition programming as problematic. For some, it occurred too far in advance of their discharge to be actionable; for others, it was compressed into the final weeks or months of service, adding stress to an already demanding period. The process itself often was described as burdensome and opaque, requiring veterans to navigate complex administrative procedures, including being redirected between offices and personnel without clear guidance. Veterans who reported more positive experiences highlighted the value of tailored programming. These included rank-specific courses, access to initiatives like SkillBridge and the Yellow Ribbon Program, and support from third-party VSOs such as Wounded Warrior Project. These resources were seen as more effective in explaining the transition process in specific terms and connecting veterans to meaningful support.

### Theme 3: Difficulties with Navigating VA System

After separation, veterans frequently encountered systemic challenges when attempting to access care and benefits through the VA. These difficulties included bureaucratic delays, poor communication, and fragmented processes that disrupted continuity of care. A particularly salient issue was the lack of in‑service medical records, such as documentation of injuries, symptoms, or treatment, which created barriers to substantiating disability claims (see Table 4 for supporting quotes).

**Table 4:** Theme 3: Difficulties with navigating VA system, subthemes with supporting quotes.

Subthemes	Frequency (n, %)	*Supporting Quotes*
**1. Frustration with VA bureaucracy, delays, communication gaps**	17 (85%)	*I did my initial disability claim while I was still in, using the BDD (Benefits Delivery at Discharge) process, and got a 90% rating. After that, I realized there were a few other things I hadn’t claimed. So, I went in and did my physical exam, but they haven’t released that paperwork to the VA to make a rating decision. That was back in November. I’ve been calling every 2–3 weeks, and they tell me something different each time: “It’s in the queue to be reviewed,” “The review process takes a long time,” “We’re going to expedite it,” “I have to email my boss to have it expedited.” It’s always something new. I keep asking, why do you need to review it? You don’t know me. You weren’t in the doctor’s office. You’ve never met me. You don’t know my military service. Just give the paperwork to the VA so they can make the decision.* -Army veteran, female, age 42
		*The other thing is they make it very difficult to even get seen by a doctor. I’ve heard stories of people who go to an appointment scheduled months in advance, because there are never open slots, and when they arrive, the doctor asks, “Did you bring these forms?” But that was never mentioned. No email, no phone call, no notice about any required documentation. Because they didn’t have some form, the appointment gets canceled and rescheduled. Basically, they just won’t see you. That kind of thing happens a lot. You’ll call to get an appointment, but the VA won’t schedule it directly. You have to talk to the contractor, or the VA will call the contractor to call you to set it up. Just nonsense. It’s a very dumb process.* -Air Force veteran, male, age 35
**2. Barriers to service due to in-service documentation gaps due to service culture**	11 (55%)	*For you to get health care or disability, you need to have a service-connected disability. But for them to determine whether it's service-connected, you need to either have a record of it, or it needs to be diagnosed while you're in the service. The problem with that is most military members are either healthy enough to just say, “F… it, it's not that bad,” or they're terrified that if they do say something, it's going to affect their work, which is a real concern. ‘Cause, let's say, “Oh, I've got a bad knee, let me go to the doc for it.” Well, now you can't go on deployment because you got put on a profile that says you have limited work duties. So, the problem is, a lot of people don't go to get things properly diagnosed or properly seen. And then by the time that they do, it's too late.* -Air Force veteran, male, age 35
		*A lot of people in the military have this mindset of, “I can't go to medical even though I'm hurt, because I have a job to do. And if I'm not there, who's going to do it?” So, it doesn’t get claimed properly while we're in. Anyone who’s already out and has gone through the disability process tries to tell people getting out: Go do all your medical paperwork. Do it properly. Go in for headaches. Go in for everything. Because when you get to these appointments, if you don’t have enough evidence that you’re struggling with something, they’re not going to take it seriously. And when you do go to these appointments, be brutally honest. I’m stubborn myself, I dealt with heart pains for almost a year before I got checked out, because I had work to do. I thought, “It’s probably nothing. It goes away in five minutes. I need to go to work. I can’t let this slow me down.” That’s the mindset a lot of people have. Then when they get out, they realize their medical file is empty, and they’re putting in claims for things that aren’t documented throughout their career.* -Navy veteran, male, age 29
**3. Support gaps and difficulties with continuity of care**	16 (80%)	*I think there were a lot of different points of failure in this, but I somehow fell through the cracks. I had a medical officer who, even though I had an MRI showing my spine was shifted and herniated discs, for whatever reason said I was fully fit for duty. It was pretty screwed up... So, I got discharged, and that ended my medical case. On paper, I looked fully fine. Because of that, I wasn’t able to get any medical help from the military. It took me about a year, but I finally got my line of duty approved. Now I can start getting treatment for an injury that happened a year and a half ago. Pretty common story I hear. I just have not been able to get help and now it's a lot worse.* -Marine Corps veteran, male, age 28
		*I wasn’t completely out, so the VA centers were kind of like, “When you retire, you can come here.” They gave me answers to certain questions, but when it came to actually doing anything, their hands were tied with certain things. It was kind of a hit-or-miss situation…The therapy piece was the biggest thing I couldn’t get done, and it took a few months. I did get a phone call from the VA. I was dealing with passive suicidal ideations, and they said they could help. They scheduled a phone appointment to try to connect me with a therapist, but when the call came, they said, “You’re not actually retired, not for a few months.” Then they said they’d call me back and it’s like, “OK, cool.”* -Army veteran, male, age 41

Veterans consistently reported significant challenges with the VA enrollment process. Many described persistent communication barriers, including difficulty obtaining clear answers to their questions and explanations of procedures. They also expressed frustration with what they perceived as unnecessary bureaucratic hurdles, such as excessive documentation requirements and complex regulations. Nearly all veterans noted long wait times for initial appointments. Once enrolled, many encountered further obstacles during verification and disability-related appointments. Several veterans highlighted how military culture, which emphasizes perseverance, minimizing personal hardship, and prioritizing the mission, often results in a lack of documented injuries or conditions, which are essential for substantiating claims. As a result of these systemic issues, veterans frequently go extended periods without receiving the care and support they need.

### Theme 4: Veteran Recommendations

Drawing from their own transition experiences, veterans offered practical recommendations to improve support systems, emphasizing the need for clearer communication, streamlined processes, and consistent health care (see Table 5 for supporting quotes). Veterans emphasized that enhancing communication during the transition from military to civilian life could significantly improve awareness of available VA resources. A commonly suggested solution was the implementation of a single point of contact or case manager, someone who could regularly check in, guide them through the process, and tailor the transition experience to their individual needs. This recommendation was especially relevant given that many veterans described the volume of information provided during transition as overwhelming, too general, and difficult to navigate. Having someone to explain and contextualize this information would be highly beneficial. Veterans also stressed the importance of continuity of care, noting that medical and mental health services received during active duty should be transferred seamlessly to the VA. Delays or gaps in care were seen as detrimental to their well-being.

**Table 5:** Theme 4: Veteran recommendations, subthemes with supporting quotes.

Subthemes	Frequency (n, %)	*Supporting Quotes*
**1. Improve communication, process transparency, and resources awareness**	18 (90%)	*Once a claim is filed and they get their rating, reaching out to help them transition for healthcare and stuff would be great. They gave me a thick book at my first appointment that tells you a lot of stuff the VA has, but I don't have time to read through that. I waited two months before going to the [VA] to get enrolled because they wouldn’t return my calls. They said, ‘We have to enroll you, you need a PCM (Primary Care Manager), and we’ll call you back,’ but nobody ever did. Just reaching out to establish care and letting people know what to do to get enrolled, what’s available, and maybe not a thick book right away. I know the VA has a lot to offer, but no one knows what that means or what it details until you have a PCM or hear from someone who says, ‘Hey, I got this from the VA.’* -Air Force veteran, female, age 35
		*I wish that there was more information on what resources are out there, and that was part of the getting-out thing. Like, they tell you a little bit about them, but you know... I guess this is true everywhere in life, but it's really, really especially true for Marines; pretty much no one actually helps anyone unless they have to. So, the mentality is very much, ‘Oh, well, you didn't go to the VA, you didn't do it on your own, so you got screwed over. That's your fault,’ kind of thing. Which I guess is true, but I don't think it should be that way. So definitely more information, and definitely more encouragement to join a group, to get into something.* -Marine Corps veteran, male, age 28
**2. Ensure continuity of care and clear handoffs**	15 (75%)	*I feel like whatever care someone is getting while they’re still active duty, in that transfer process should already have a place to go, for it to be transferred and continued. To completely cut it off and not have it set up when they’re getting out is wrong. If someone struggles with PTSD or other mental health issues, and you just cut them off and give them the boot – this is someone who probably wanted to do 20-plus years in the military but can’t – it’s no wonder suicide rates are as high as they are for veterans. You just drop them off in the middle of nowhere.* -Navy veteran, female, age 31
		*If they were able to find a way to have continuity of care between the losing facility and wherever someone is going, that in-between mental health care would be really great. Whether it's just counseling, it doesn't have to be a psychologist or psychiatrist, but some kind of something. Just having some kind of continuity of care while waiting to be established would be great. Because if a veteran doesn't have a support system at all, or they're knee-deep and bad enough to be separated or retired from the military due to mental health, that could be extremely detrimental, not having an outlet while they're waiting.* -Air Force veteran, female, age 35
**3. Normalize and mandate mental health and adjustment support**	10 (50%)	*I’ll say that the preparation for how lonely it’s going to feel for probably the first couple of months is important. When you see the world moving on and you’re just still there, that’s how it felt for me. I’ll say the first couple of months, not knowing what I was gonna do, kind of feeling stagnant in that way. The mental let-downs of just not knowing what might happen is definitely something people should be prepared for – how the separation feels and going away from this environment you’ve known to a new one…I think people should probably get a therapist when they get out. They should make that a mandatory thing.* -Marine Corps veteran, female, age 25
		*I just wish there was something, somewhere along the line, that kind of prepared you for the highs and lows. Like, ‘Hey, you're gonna do good. You're gonna be successful. But also, just get ready to be depressed for a bit. You’re gonna get out, and somehow you're gonna lose touch with all your friends, because they're all getting out too.’ I just feel like there needs to be something preparing you for that part. You're getting out. It's supposed to be, you know, separation from the military. It should feel more like a loss of a job, or moving on from a job, than moving on from a whole lifestyle. Maybe just more of a warning, like, ‘Hey, this is gonna be like nothing you've ever experienced before. Be ready to be sad.’* -Marine Corps veteran, male, age 26

Veterans also called for normalization of emotional adjustment support, recognizing the psychological toll of leaving military life. Current transition programming tends to focus narrowly on career planning and financial literacy, while neglecting the psychological and cultural shifts that accompany separation from military life. Many veterans described feeling unprepared for the loss of structure, community, and purpose, and expressed a need for therapeutic support to help navigate this major life change.

## Discussion

This study aimed to explore veterans’ transition experience during their process of separating from the military, including their perceptions of the barriers to care, how they navigate the various systems which offer support, and recommendations for improvement. Importantly, while there are numerous programs and services in place to support veterans during the transition, veterans face persistent systemic barriers, revealing a critical need for personalized, coordinated, and psychologically responsive support systems. Our analysis revealed four major themes, which together illustrate how structural, procedural, and cultural factors influence veterans’ transition outcomes.

The first theme revolved around the complex interplay of physical, mental and bureaucratic stressors during discharge from service. Consistent with prior literature on military-to-civilian transitions [29,30], participants described discharge as a period marked by deteriorating health, emotional distress, and procedural confusion. Veterans with musculoskeletal disorders often face premature or medically-driven discharge, frequently under conditions that do not allow for adequate preparation or planning [31–33]. Chronic pain, mobility limitations, and functional impairments compound the emotional and procedural stressors of separation and may heighten vulnerability to poor post-discharge outcomes. These transitions are compounded by procedural barriers, such as delays and lack of transparency, that disproportionately affect those with urgent medical needs [34]. Prior research has shown that veterans discharged due to injury or medical conditions often face more difficult transitions, including barriers to employment, social reintegration, and access to care [35–37]. Taken together, these findings suggest that systemic obstacles begin well before veterans engage with post-separation services, potentially cascading into further difficulties navigating VA care and benefits.

The second theme reflected mixed perceptions of transition-related programming. Many participants described the process as perfunctory, designed more to “check a box” than to offer meaningful support during a critical period. Veterans frequently characterized the programming as “generic,” noting a lack of tailored resources or a mismatch between their individual needs and the support provided. This aligns with critiques of TAP as insufficiently tailored to individual needs [38]. Further, although transition programming has received considerable attention and undergone multiple refinements over the past decade, veterans’ reported a disconnect between program design and individual needs. Notably, there is a lack of robust data evaluating the effectiveness of these programs [13,39], making it difficult to determine whether recent changes have translated into improved outcomes. This gap is further complicated by the fact that many transition-related programs are created or sustained through congressional mandate rather than evidence-driven design, limiting DoD and VA flexibility to refine or streamline them even when misalignment with service member needs becomes apparent. Without systematic evaluation and greater adaptability, resources may continue to be directed toward efforts with limited utility while more impactful, veteran-centered supports remain underdeveloped.

The third theme revealed persistent systemic barriers in accessing care and benefits post-separation. Veterans described bureaucratic delays, fragmented processes, and poor communication as major obstacles, particularly during the early months of reintegration. These barriers are consistent with previously documented barriers related to organizational problems, such as prolonged wait time, which doubles the likelihood of a veteran not returning to receive care at the VA [17,40,41]. These delays not only hinder access to essential services but also exacerbate existing health conditions, particularly for those with urgent physical or mental health needs [1]. For veterans with musculoskeletal conditions, delays in accessing care can exacerbate pain, reduce mobility, and contribute to psychological distress [34,42]. The lack of timely rehabilitation services or continuity in pain management may lead to worsening disability and disengagement from care systems.

A key barrier identified was the lack of in-service medical records, often driven by military cultural norms that discourage help-seeking and prioritize mission over self [43–45]. Many veterans withheld health concerns during service and at discharge, often due to fear of career consequences. These dynamics vary across communities and operational roles, with personnel in readiness-critical or safety-sensitive fields often facing heightened disincentives to disclose symptoms or seek care. Although mandated Periodic Health Assessments are intended to capture emerging health issues, their utility is limited when service members are reluctant to report conditions due to fears of duty restrictions, deployment loss, or adverse impacts on career progression. This underreporting creates challenges when Veterans file claims for service-connection compensation; award of these claims are crucial determinants of what services veterans are eligible for. Service-connection claims that are denied due to lack of documented symptoms while in the service also limit the reach of transition programs like *in*Transition, which rely on documentation or referrals to identify those in need. As a result, high-risk individuals may be excluded from early support. Future research examining the mechanisms that drive nondisclosure across career fields would complement the present findings and clarify how structural and cultural factors jointly shape reporting behaviors.

These findings also underscore differences between DoD and VA approaches to musculoskeletal care. DoD’s return‑to‑duty model emphasizes mission readiness and rapid recovery, whereas VA uses a biopsychosocial, whole‑person framework focused on early intervention and pain management. Military norms of pushing through pain may reduce opportunities for early treatment, increasing the risk that acute injuries become chronic. To address these gaps, transition programs must recognize cultural barriers to disclosing illness while in-service and offer other accessible options for veterans to connect with care. Existing efforts, such as the “Don’t Wait. Reach Out.” and “Make the Connection” campaigns [46,47], and peer support initiatives like VA Peer Specialists [48] aim to reduce stigma and promote help-seeking. These culturally informed interventions are promising, but broader structural reforms, such as improved coordination between DoD and VA and policies that encourage safe disclosure, are needed to ensure all veterans receive timely, appropriate care.

Finally, the fourth theme offers a roadmap for improving transition systems. Veterans emphasized the importance of clearer communication, continuity of care, and emotional adjustment support. The recommendation for a single point of contact or case manager reflects a desire for relational continuity and personalized guidance, which are elements shown to improve engagement and outcomes in other healthcare settings [49–51]. Two such individualized health care access programs exist, namely, VA’s National Liaison Program and the Post-9/11 Military2VA (M2VA) Case Management Program. Both programs focus on early identification of active‑duty service members with ongoing health‑care needs and provide care coordination and case management as they move from DoD installations to VA health care [52]. The VA Liaison Program has 48 VA Liaisons for Healthcare onsite at military treatment facilities and regionally (including worldwide installations) to provide education individualized to the transitioning service member’s health care needs and clinically coordinate ongoing care at VA facilities nationwide. Post-9/11 M2VA screens Post-9/11 era veterans for high-risk needs and assigns case managers at VA facilities to help veterans navigate benefits, connect with health care providers, and ensure continuity of care across systems. This model directly addresses many of the concerns raised by participants in this study, particularly around fragmented communication and gaps in support. Veterans managing chronic musculoskeletal conditions expressed a need for consistent, coordinated care that addresses both physical and psychological dimensions of disability. Programs like the National VA Liaison and Post-9/11 M2VA Programs are particularly beneficial for this group, ensuring continuity in pain management, rehabilitation, and mental health support. Adequately funding, expanding, and adapting programs like Post-9/11 M2VA to reach a broader population of transitioning veterans could be a key strategy in improving outcomes and reducing the sense of abandonment often described during this critical period.

Veterans in this study also emphasized the need for emotional support during transition, particularly in relation to identity disruption and the loss of purpose. These concerns are well-supported by a growing body of research demonstrating that unresolved shifts in identity and meaning following military separation are associated with poorer mental health, diminished physical well-being, and challenges in social reintegration [53–57]. Despite this evidence, current transition programming remains primarily focused on logistical domains such as employment, financial literacy, and benefits navigation, with limited attention to the psychological and existential dimensions of separation [58]. Interventions that explicitly address identity may show promise in improving adjustment and long-term outcomes [59–61]. For veterans with chronic musculoskeletal disorders, the loss of physical capability often intersects with identity disruption, compounding the emotional toll of transition [57]. Importantly, our findings highlight that musculoskeletal pain is not solely a physical phenomenon; by definition, pain is both a sensory and emotional experience processed through brain systems tightly linked to emotion and threat. Thus, worsening physical pain, mental health strain, and identity disruption during transition are not independent processes but mutually reinforcing ones, amplifying vulnerability during discharge. Addressing these intertwined challenges requires psychologically responsive systems that recognize the lived experience of chronic pain and disability. Without intentional integration of identity-focused care, veterans may continue to encounter unmet needs during a period marked by vulnerability and change.

### Limitations

Several limitations should be acknowledged when interpreting these findings. First, although the sample was drawn from a national pool and intentionally selected to enhance diversity, its size was relatively small. This is common in qualitative research but may constrain the generalizability of results to the broader veteran population. Additionally, some participant groups were intentionally oversampled to ensure adequate representation, which may further limit the generalizability of findings to the broader veteran population. Second, the focus on veterans with musculoskeletal disorders narrows the scope of applicability, making it less relevant to those without such conditions. Third, all participants had interacted with the Veterans Benefits Administration to file claims, which may skew the findings toward individuals already connected to VA systems and exclude perspectives from those who remain outside these networks. Broader sampling that includes veterans without musculoskeletal conditions and those outside VA systems will enhance generalizability and identify subgroup-specific needs. Additionally, we were not able to explore possible differences in reintegration experiences across discharge types or claim submission pathways (e.g., BDD versus other timelines), and future research could examine whether these factors shape Veterans’ perceptions. Fourth, as with most qualitative studies, self-selection bias is possible; participants who volunteered may differ in meaningful ways from those who did not, potentially shaping the themes identified. Fifth, the cross-sectional nature of the study limits the ability to draw conclusions about changes over time or causal relationships. Future research should employ longitudinal, mixed methods designs to track veterans from pre-separation through multiple years post-discharge, examining how early barriers, such as documentation gaps and bureaucratic delays, shape long-term health, functional, and psychosocial outcomes. Sixth, this study did not specifically focus on the enterprise-wide programs within VHA that focus on health care education and coordination in concert with benefits delivery. Finally, concentrating on veterans within one year of separation provides valuable insight into early transition experiences but does not capture longer-term perspectives that might reveal how veterans adjust over time.

### Future directions

System-level and cultural changes are critical for improving engagement. Stigma remains a major barrier to care-seeking in military populations [62–64]. Cultural interventions, such as peer support programs and stigma-reduction campaigns, can foster trust and normalize help-seeking [65]. Policies that enable safe disclosure during service are essential for reducing fear of negative consequences, as confidentiality concerns often deter service members from reporting mental health issues [66,67]. Both require scale-up studies to determine how widely they can be implemented and whether they remain effective when expanded beyond small pilot settings. Separately, structural reforms like improving DoD–VA data sharing should be prioritized to enhance continuity of care [68]. Technological solutions, including automated health record transfers and pre-scheduled care handoffs, should also be evaluated for their impact on timeliness and coordination. For example, VA’s Electronic Health Record Modernization (EHRM) initiative, which aims to provide a single, longitudinal record across DoD and VA paused new deployments in 2023 due to patient safety and performance concerns [69,70]. Six medical centers currently are live with the new EHR, and 13 additional facilities will deploy it in 2026; comprehensive assessment alongside these deployments is essential to ensure interoperability with DoD systems and sustained clinical benefits [69,70].

Rigorous evaluation of transition programs is also essential, not only to determine effectiveness but also to understand why existing resources remain underutilized and some programs (i.e., VA Liaison and Post-9/11 M2VA Case Management) are not scaled for the entirety of the transitioning service member and Post-9/11 era veteran population. Documented barriers include lack of awareness among veterans and providers, unclear eligibility criteria, staffing shortages, and logistical challenges in enrollment [71,72]. Advancing research into these barriers through mixed-methods research that combines utilization data with qualitative interviews could inform strategies to improve program visibility, accessibility, and capacity. Implementation research should build on these insights by developing and testing scalable strategies that not only promote program coordination and continuity of care but also directly address identified barriers to program uptake, such as awareness gaps, staffing limitations, and enrollment challenges, to ensure that existing resources are effectively utilized.

Finally, mental health care should be more formally integrated into transition programming. Evaluations of integrated approaches pairing logistical support with psychologically responsive care are needed to address compounded challenges for veterans with chronic pain and disability. These directions aim to advance transition systems from generic programming toward personalized, coordinated, and culturally attuned models that address physical, psychological, and structural determinants of successful reintegration.

## Conclusion

This study underscores the challenges of the military-to-civilian transition, particularly for veterans managing service-connected musculoskeletal conditions. Despite the existence of structured programs and resources, systemic barriers such as bureaucratic delays, long wait times, fragmented communication, and cultural norms that discourage help-seeking continue to impede access to timely and coordinated care. Veterans’ recommendations highlight the need for personalized, psychologically responsive support systems that prioritize continuity of care and emotional adjustment alongside logistical planning. Expanding case management models, improving transparency, and integrating identity-focused interventions could help bridge critical gaps and reduce the sense of abandonment often described during this vulnerable period. Ultimately, addressing these structural and cultural challenges is essential to promoting successful reintegration and improving long-term health and well-being for transitioning veterans.

## Supporting information

S1 Appendix Qualitative Interview Guide: Exploring Identity Challenges in Recently Separated Veterans This file provides the interview guide for interviews conducted for this study. (DOCX)

S2 Appendix Integrated Reporting Framework Mapping: PARRQA, COREQ, SRQR, and NICE. This file provides an integrated mapping of rapid qualitative rigor criteria (PARRQA) and qualitative reporting guidelines (COREQ, SRQR, NICE) to the study procedures described in the manuscript. Items are consolidated to avoid redundancy, with each domain mapped to the relevant section(s) of the Methods.
(DOCX)
