# Supplementary material for: Bridging the gap: A qualitative study on veterans’ experiences with systemic barriers and support during transition
Source: PLoS One. 2026 Nov 6;21(11):e0361285. doi: 10.1371/journal.pone.0361285 (PMC13742960; doi:10.1371/journal.pone.0361285)
Supplement: Supplementary file 1 [file pone.0361285.s001.docx]

**S1 Appendix.**

**Qualitative Interview Guide: Exploring Identity Challenges in Recently Separated Veterans**

***********IN THE MILITARY

“Because the military brings together people from different backgrounds and helps them develop shared attitudes, values, and expectations of military culture, I want to start first by getting a sense of what being in the military was like for you.”

1. I’d like to gather more details about some specifics about your service. Tell me about your service in the military (what branch, job, rank, how long did you serve, deployments).

2. How would you describe yourself [in terms of your personality, key characteristics, ways of relating to others] when you were in the military?
Prompt: How much of that did you learn from the military and how much of that was part	of you or the way you saw yourself before being in the military

2a. How would you describe how you related to/got along with others when you were in the military?

***********DISCHARGE PHASE

“Let's move on to the next phase of your journey, which is your discharge from service. I’m going to ask you about 2 different parts of the transition: preparing for discharge and the discharge process and then the transition piece/claims process.”

3. What type of discharge did you receive (voluntary/unvoluntary; honorable, general, other, medical)?

4. What assistance, if any, did you receive while in the military to plan for your discharge and transition to civilian life?

5. When you were preparing for discharge, what did you think returning to civilian life would be like? OR what did you think life would be like when you returned to civilian life?

5a. How did you think civilians were viewing you/were going to view you when you first got discharged from service?

**********POST DISCHARGE

“When someone transitions from a role that’s been important to them or that they’ve held for a long time, like being in the military, they may struggle to define themselves outside of that role. I want to explore what life has been like for you since discharging and leaving the military.”

6. So far, we’ve discussed that, in the military, you saw yourself [INSERT SUMMARY OF WHAT THEY SAID]. Please describe how you see yourself now, after leaving the military. What changes have you noticed in how you view yourself now compared to your military days?

6a. How have these changes, [DESCRIBE CHANGES], impacted your transition to civilian life?

7. In what ways do you think your experiences in the military have shaped you differently from someone who has never served? How has this affected how you fit in with people who have not served/civilians?

8. You’ve filed a claim for service-connected injury. In what ways does filing the claim or going through the claims process reflect how you view yourself? What about having an injury?

9. How have your personal struggles since leaving the military — such as loss of sense of who are you are, difficulties managing military and civilian roles, loss of purpose or meaning [OR SUMMARIZE WHAT WAS SAID ABOVE] - impacted your interests in your occupation, home-related activities, and hobbies?

10. How have your personal struggles since leaving the military — such as loss of sense of who are you are, difficulties managing military and civilian roles, loss of purpose or meaning [OR SUMMARIZE WHAT WAS SAID ABOVE] - impacted your interpersonal relationships with family, friends, and significant others?

11. How involved are you in community life, such as clubs, churches, or veteran’s organizations? How have your personal struggles since leaving the military — such as loss of sense of who are you are, difficulties managing military and civilian roles, loss of purpose or meaning [OR SUMMARIZE WHAT WAS SAID ABOVE] - - impacted your involvement in the community?

12. What things (skills, qualities, strengths) do you think you gained in the military that are helping you in the civilian world?

13. Are you currently enrolled in the VA? If not, are you planning to do so? Why or why not? How do you think the VA could be helpful to you during this transition?

14. Considering the challenges you have been experiencing since exiting the military, what specific strategies or support would you find most beneficial in navigating this transition? Are there particular resources or forms of assistance that you believe could be helpful?

Prompt: Additionally, would you find counseling or similar support helpful in coming to terms with the changes and challenges of this transition?

15. Is there anything else about the challenges you experienced in how you see yourself or the roles you serve during the transition from military to civilian life that we’ve not discussed that you want to share?
