# Supplementary material for: Bridging the gap: A qualitative study on veterans’ experiences with systemic barriers and support during transition
Source: PLoS One. 2026 Nov 6;21(11):e0361285. doi: 10.1371/journal.pone.0361285 (PMC13742960; doi:10.1371/journal.pone.0361285)
Supplement: Supplementary file 2 [file pone.0361285.s002.docx]

**S2 Appendix
Integrated Reporting Framework Mapping: PARRQA, COREQ, SRQR, and NICE**

This supplementary file provides an integrated mapping of rapid qualitative rigor criteria (PARRQA) and qualitative reporting guidelines (COREQ, SRQR, NICE) to the study procedures described in the manuscript. Items are consolidated to avoid redundancy, with each domain mapped to the relevant section(s) of the Methods.

| **Domain / Item** | **Framework(s)** | **Location in Manuscript** | **How Addressed in This Study** |
| --- | --- | --- | --- |
| Research question, purpose | PARRQA, SRQR | Introduction; Study Approach & Design | Purpose and applied focus are stated, emphasizing post-separation experiences and systemic barriers. |
| Methodological orientation / Rationale | COREQ, SRQR, NICE | Study Approach & Design | Pragmatic orientation and rationale for rapid analysis described. |
| Definition and justification of rapid qualitative analysis | PARRQA | Study Approach & Design | Rapid qualitative analysis defined and justified. |
| Use of theory/framework | PARRQA, SRQR | Study Approach & Design; Analysis | Domains guided data collection and matrix organization. |
| Sampling strategy, inclusion criteria, recruitment | COREQ, SRQR, NICE | Participants & Recruitment | Purposive sampling; inclusion criteria; recruitment outcomes reported. |
| Researcher roles, training, reflexivity | COREQ, SRQR, NICE | Interviews & Data Collection; Analysis | Lead author conducted interviews; field notes; reflexive discussions documented. |
| Relationship with participants | COREQ | Interviews & Data Collection | No prior relationship; role explained during consent. |
| Ethical approval, consent | SRQR, NICE | Participants & Recruitment; Interviews & Data Collection | VA IRB-approved; verbal consent obtained. |
| Data collection setting and procedures | COREQ, SRQR, NICE | Interviews & Data Collection | Virtual Teams interviews; 45–60 minutes; pilot-tested guide. |
| Field notes/documentation | COREQ, SRQR | Interviews & Data Collection | Detailed notes taken using standardized template. |
| No repeat interviews; transcript return | COREQ | Interviews & Data Collection | No repeat interviews; transcripts not returned due to rapid design. |
| Data handling and security | SRQR, NICE | Interviews & Data Collection | Data stored on VA-secure encrypted servers. |
| Summary templates and data linkage | PARRQA | Interviews & Data Collection | Standardized templates; audio timestamps maintained. |
| Matrix analysis and software | PARRQA, SRQR | Analysis | Excel used for matrix, comparison, and audit trails. |
| Analyst team and consensus | COREQ, SRQR, NICE | Analysis | Four analysts; iterative meetings; consensus refinement. |
| Theme derivation (deductive + emergent) | COREQ, SRQR | Analysis | Themes refined using deductive structure with emergent insights. |
| Saturation / Thematic sufficiency | SRQR | Analysis | No new domains in final interviews; sufficiency achieved. |
| Trustworthiness techniques | SRQR, NICE, COREQ | Analysis | Triangulation, consensus, audit trails, timestamp verification. |
| Member checking | COREQ | Analysis | Not conducted due to confidentiality and rapid design; alternatives used. |
| Participant characteristics reporting | COREQ, SRQR | Participants & Recruitment | Demographics and service characteristics fully reported. |
| Use of illustrative quotations | COREQ, SRQR, NICE | Results | Quotes included in thematic tables. |
| Integration with prior work | SRQR | Discussion | Themes contextualized with transition literature. |
| Limitations and transferability | SRQR, NICE | Limitations | Study limitations related to sampling, design, timing addressed. |
| Transparency of reporting standards | SRQR, COREQ, NICE | Analysis | Manuscript states alignment with all reporting frameworks. |
